# Cytogenetic investigation of couples with recurrent spontaneous miscarriages

**DOI:** 10.12669/pjms.35.5.678

**Published:** 2019

**Authors:** Misbah Iqbal Hanif, Ayesha Khan, Afsheen Arif, Erum Shoeb

**Affiliations:** 1 Misbah Iqbal Hanif, MS. Department of Genetics, University of Karachi, Karachi, Pakistan; 2 Prof. Ayesha Khan, MBBS. MRCOG. FRCOG, Department of Gynecology and Obstetrics, Civil Hospital, Karachi, Pakistan; 3 Dr. Afsheen Arif, PhD. Dr. A.Q Khan Institute of Biotechnology and Genetic Engineering, University of Karachi, Karachi, Pakistan; 4 Dr. Erum Shoeb, PhD. Department of Genetics, University of Karachi, Karachi, Pakistan

**Keywords:** Marker chromosome, Robertsonian translocation, Reciprocal translocation, Inversion

## Abstract

**Background & Objective:**

Spontaneous pregnancy loss has always been the frustrating experience for the couples and concern clinician. Chromosomal abnormality in either of the parent is considered to be the one of the leading cause of recurrent spontaneous miscarriages. This study was designed to evaluate the possible chromosomal etiology of miscarriage and the subsequent intimacy of maternal or paternal genetic abnormality.

**Methods:**

This case-control study was conducted between January 2016 and October 2016 at a tertiary care hospital in Karachi. A total of thirty-two couples were selected who had suffered with recurrent spontaneous miscarriages (RSM). Using conventional cytogenetic technique karyotyping was performed on all of the subjects. For the control twenty couples were also selected with no history of pregnancy loss. All the karyotypes were recorded on the standard method. Data was analyzed through SPSS version 22.

**Results:**

Among thirty-two cases nine cases were found to have abnormal karyotype. In which sex chromosomal trisomy=02 (46,XY/47,XXY), marker chromosome=01 (47,XX,+mar), Robertsonian translocation=01 (45,XY,der,(14:21),(q10;q10)), reciprocal translocation=01 (46,XX,t(11;22)(q23;q11.2)), inversion=02 (46,XX,inv(9)(p11q13)) and minor structural abnormalities=02 (46,XX,15PS+) were found. Approximately equal ratio with 1:1.25 was observed among male and female carrier respectively. Non-significant difference was found between the ages of both carriers (p=0.34). Though a significant different value was calculated in the case of number of miscarriage (p=0.004*). Moreover, no significant association was found among spontaneous miscarriage (SM) and recurrent spontaneous miscarriage (RSM) with respect to maternal age (p= 0.157).

**Conclusion:**

In the recent study possible chromosomal abnormalities suggested the evaluation of the patient with the history of recurrent spontaneous miscarriage must include conventional cytogenetic. Moreover, probe development and extended investigation can ease the prognosis among pregnancy related complication.

## INTRODUCTION

Pregnancy loss is always destructive for the couple and their clinician. Spontaneous miscarriage (SM) is the most common dilemma of the reproductive event without any prior history or reason, comes about 1-3% of all women of reproductive age. Even so the intensity of grief reaches its peak if it happens repeatedly as in the case of recurrent miscarriages.^1^ Fetal development is multifarious process which requires equilibrium of all hormonal environmental and genetic factors, abnormality in either factor can lead in the abnormal development of embryo or fetal loss. Spontaneous miscarriage is actually nature’s quality control for selecting genetically normal offspring. Though, it is always devastating for the couple who suffers with miscarriage. Spontaneous miscarriage (SM) is defined as the loss of an embryo before 20^th^ week of gestation or if the fetus loss with less than 400g of weight. Spontaneous miscarriage (SM) is the most familiar complication of pregnancy at the rate15-20% of all quantifiable pregnancies.^2^,^3^

Recurrent spontaneous miscarriage (RSM) is described as two or more consecutive pregnancy losses before the 20th week of gestation, though it over all distresses 3% of the couples attempting to begin a family.^4^ The etiology of RSM is often uncertain and may have several factors, with a great extent of debate regarding diagnosis and treatment. Some of the established causes include; anatomical, immunological, endocrine, infectious, nutritional, environmental and genetic factors. Despite the fact, 99% of all clinically recognized pregnancy with chromosomal abnormality ends up with miscarriage.^5^ These chromosomal abnormalities can be numerical as well as structural. Most of the numerical chromosomal abnormalities are caused by the non-disjunction during gamete formation. In case of RSM 3-5% of couple’s one partner must have reciprocal translocation or balanced chromosomal rearrangements and are carriers of chromosomal abnormalities and transmits to their offspring.^6^

Numerical chromosomal abnormalities are more frequently found in the product of conception in case of SM, includes trisomy, monosomy and polyploidy. However, chromosomal heteromorphism is more important during the investigation of couples with RSM (Term heteromorphism is used to define the normal variant of karyotype or synonymously with polymorphism).^7^ Similarly chromosomal translocation or rearrangement with no overall gain or loss is also reported as the reason of RSM or infertility. Chromosomal heteromorphism and the translocation can be observed through G-banding technique.^8^ Presence of inversion or translocation among the couples is not only associated with RSM but increase the risk of giving birth of a child with congenital defects.^9^

Prenatal cytogenetic analysis could be beneficial for the couples having RSM to rule out the possible rearrangement of chromosome consequently genetic counseling. Cytogenetic investigations have been the gold standard technique for decades in several countries to investigate pattern of chromosomal anomalies among the couples having RSM. Chromosomal deletions, duplications, inversions, reciprocal and Robertsonian translocations, all are related with RSM.^10^ Even though many of the genetic abnormalities are *denovo* but it can greatly reduce the anxiety and grief of the couple with RSM of being incomplete.^5^

Routine prenatal cytogenetic testing is still uncommon practice in our society. This regrettable fact of our society has an impact on the management of RSM. The aim of this study was to evaluate the frequency of chromosomal abnormalities in couples having SM and RSM in Karachi, Pakistan. This study was undertaken to emphasize the over sighted fact of RSM and thereby contribute to the literature in context of chromosomal abnormalities.

## METHODS

The presented case-control was conducted at the department of obstetrics and gynecology, Civil Hospital, Karachi, Pakistan, a tertiary referral center serving 1900 bed, affiliated with Dow University of Health Sciences after the approval of Institutional Ethical Review Board. A total of 52 couples and 186 women (243 cases) were investigated during the study period between January 2016 and October 2016. Among this 32 couples were selected as the patient group who had suffered with recurrent spontaneous miscarriage and underwent of the D&C (dilation and curettage). All the enrolled patients had no identified causes for abortions. The second group comprised of 20 healthy couples with no history of miscarriage or intra-uterinal death, and recently gave birth to a normal child; this group was represented as the control group.

The inclusion criteria were any patient having first trimesteric RSM and the exclusion criteria were any patient with any possible cause of this RSM, including uterine abnormality, hormonal imbalance (especially history of thyroid dysfunction), clinical profile with antibodies of cardiolipin, phosphatidyl serine, lupus anticoagulant, Rh factor or any recent infection. Conventional cytogenetic was performed on the blood samples of couples to rule out the possible chromosomal abnormality more precisely chromosomal rearrangement. Every patient was interviewed and consent was taken from the couples. Karyotyping was executed by using conventional cytogenetic technique, for the purpose 5ml of peripheral blood sample was collected in sodium heparin test tubes. Cell culturing was executed in RPMI cell culture medium with FBS (Fetal Bovine Serum) at 37°C for 72 hours. Metaphase chromosomes were arrested through Colcemid addition for 30 minutes followed by hypotonic KCl treatment for one hour and later fixation was done by using 3:1 methanol-acetic acid mix. G-banding was performed by Geimsa and Trypsin treatment; according to standard method.^3^ Karyotypes were recorded according to the recommendations of ISCN, 2013.^11^

All data were analyzed using SPSS Version 22. Parametric variables were compared using the Independent Sample *t*-tests or the Chi-square test. All results are reported as means ± SD or number (percentage). P < 0.05 was considered to be statistically significant.

## RESULTS

**Fig.1:**
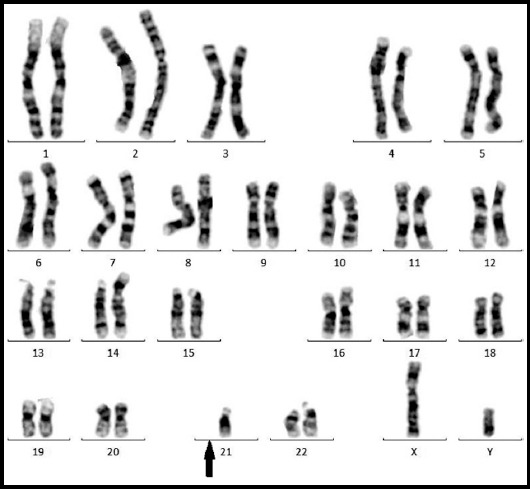
A Karyotype of the male with Robertsonian translocation between chromosomes no. 14 and no.21, 45,XY,der,(14:21),(q10;q10).

**Fig.2:**
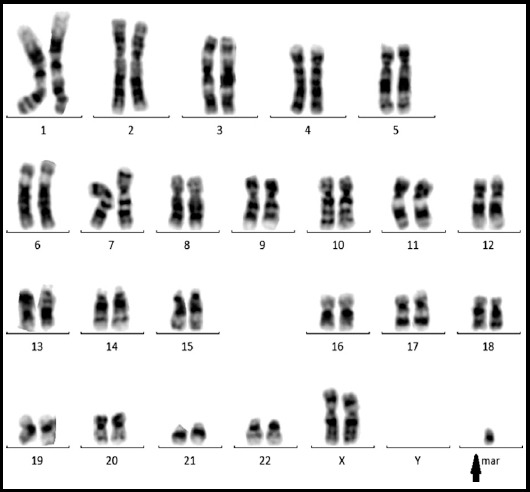
A Karyotype of the female with an extra Small supernumerary marker chromosomes, 7,XX,+mar.

In subject group of 32 couples were recruited with the history of recurrent spontaneous miscarriages, and 20 healthy couples were included as the control group. Conventional cytogenetic analysis was carried out, and numerical and structural abnormalities were detected among nine cases (28.12%). Of these four were male carriers; two cases of trisomy, one Robertsonian translocation (shown in Fig.1; translocation among chromosome 14 and 21) and one case of increase in length (structural change), and five were female carriers two inversions, one marker chromosome (shown in Fig.2; female karyotype with an extra marker chromosome), one translocation and one case of increase in length (structural change), all of these karyotypes has been shown below. The partners of these 9 carriers had normal karyotype. Patients were recruited randomly from the single center, all of the enrolled patients belonged to low socioeconomic status and different ethnic background. The consanguineous ratio among control was approximately equal, though among the recruited subjects 62.5% were cousins. The ratio of males/females was approximately equal 1:1.25 and found to have variety of karyotypes among both genders, as shown in Table-I. Additionally no chromosomal abnormality was found among the control group individuals.

**Table I:** Karyotype, Age and No. of Miscarriage.

Gender	Karyotypes	Age	No. of Miscarriages	Chromosomal Abnormalities
Male	46,XY(07)/47,XXY(09)	31	3	Trisomy
	46,XY(08)/47,XXY(05)	40	4	Trisomy
	46,XY,15PS+(15)	33	3	Minor Structural Abnormality
	45,XY,der,(14:21),(q10;q10)	36	7	Robertsonian Translocation
Female	46,XX,15PS+(19)	40	3	Minor Structural Abnormality
	46,XX,inv(9)(p11q13)(17)	29	4	Inversion
	46,XX,inv(9)(p12q13)(15)	30	3	Inversion
	46,XX,t(11;22)(q23;q11.2)	34	3	Reciprocal Translocation
	47,XX,+mar	28	3	Marker

Total no. of cases = 09, Data analyzed by Chi-square test and the value <0.05 reported as non-significant.

The comparison of age and the number of abortions between male and female carriers is shown in Table-II Although there were no statistical difference in the ages of the female and male carriers (p value>0.05), the number of abortions in the case of male carriers were significantly higher than that in the case of female carriers (p value <0.05).

**Table II:** Comparison of age and No. of Miscarriage.

Gender	Male Carriers	Female Carriers	P
Age	35.0 ± 3.91	32.2 ± 4.91	0.34
No. of Abortions	4.25 ± 1.89	3.2 ± 0.45	0.004*

Data are reported as means ± SD, Data were assessed by the Independent Sample t-test, as appropriate p-value<0.05.

The frequencies of miscarriages among SM and RSM groups were also evaluated through chi-square analysis and p value=0.157 were found to have non-significant (p value >0.05) at different group of ages, illustrated as graphical representation.

**Graph.1:**
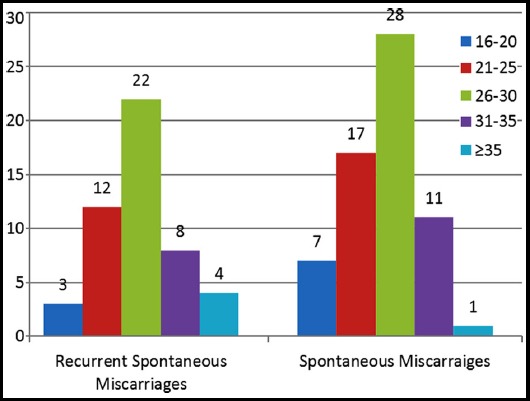
Comparison of SM and RSM in different maternal ages. **Graph:** Number of events of miscarriage between SM and RSM was compared with advancing maternal age by Chi-square, and (p value= 2.00) was found to be non-significant.

## DISCUSSION

Alteration in chromosome number or structure is well known reason of recurrent spontaneous miscarriage or neonatal death. In current study overall prevalence of chromosomal defects was found to be almost equal in both genders with 55.6% in female and 44.4% in male, with no statistical difference of abnormal karyotype (p value>0.05). This finding is supported by Goud 2009, as they have reported similar frequencies in either gender participated in RSM.^4^

The occurrence of chromosomal abnormality was reported higher in female partner among the couples with recurrent pregnancy. A possible reason of this mechanism is the production of single ovum each month. However millions of sperms release in every expulsion, so the nature select against the abnormal gametes.^10^

In this case-control chromosomal abnormality was found to be 28.12% with 18.75% structural chromosomal abnormalities followed by numerical abnormalities at 9.3%. In first two couples 46,XY/47,XXY mosaicism were found in their male partner, these couples had three recurrent spontaneous miscarriage. Several studies reported the involvement of sex chromosome trisomy in RSM.^12^-^14^

Two cases were encountered of increased in the length of chromosome 15 p arm, one of them were male carrier and second were female. This polymorphism can be related with recurrent miscarriage RSM as it is acquiring additional structure (large satellite on chromosome 15), which has the tendency to lead abnormal chromosomal segregation during meiosis; though it can be observed apparently as normal individual. Additionally ps+ on chromosome 15 with RSM is more commonly reported in male carriers however, in the recent data equal number of chromosome 15 heteromorphism were found in both genders.^1^,^12^,^15^

A male carrier of Robertsonian translocation (chromosome 14;21) was also reported, this couple had suffered with seven recurrent miscarriages. Some of the researchers suggested female carrier of Robertsonian translocation are more prone to have RSM though our finding disclose the importance of Robertsonian translocation in male carrier.^4^ Hasanzadeh-NazarAbadi M et al., in 2014 stated the involvement of Robertsonian translocationof 14;21 in recurrent miscarriage.^16^ In Robertsonian translocation carrier can be phenotypically normal nevertheless, have the probability of passing genetically abnormal gametes which can lead to miscarriage.

Presented results revealed a female carrier of reciprocal translocation between chromosome 11;22 with three miscarriages. Similar translocation between 11;22 has been reported in a case study by Jobanputra V et al., 2005 and Correll-Tash Set al., 2018. Female with 46,XX,t(11;22)(q23;q11.2) karyotype had suffered with 8 miscarriages in her first trimester.^17^,^18^ Even though Ghazaey S et al., in 2015 has reported male carrier of this translocation with repeated history of miscarriage.^12^ In balanced reciprocal translocation switching of chromosomal fragments occur which leads to structural chromosomal rearrangements. Moreover, the size of the exchanged segment matters the degree of severity.^5^ In many studies it was stated that the reciprocal translocation is one of the leading cause of recurrent spontaneous miscarriage among the apparently healthy individual.^5^,^12^,^15^

The current study disclosed two cases of chromosome nine inversions both of them were female partner and had suffered with three RSM. Several studies have supported chromosome nine inversion involvement in recurrent miscarriages. It is the most common heteromorphism observed in general population with the complaint of RSM and birth defects. Carriers of chromosome inversion nine are more labile to produce unbalanced gametes therefore at a risk of having an offspring with unbalanced karyotype.^4^,^7^,^12^,^15^,^19^

In the last case of Table-I female carries a marker chromosome, with the history of three miscarriages. Ghazaey S et al., in 2015 repoted 19 cases of marker chromosome associated with RSM.^12^ Small supernumerary marker chromosomes are defined as additional centric chromosome fragments which are excessively small to be identified through cytogenetics alone. The association of supernumerary marker chromosome in RSM has been previously reported could be due to a partial trisomy of some genes.^20^,^21^

The mean maternal age of the subjects carrying anomalies was 32.2 and paternal age was 35 years, Mozdarani H et al., in 2008 and Ayed Wet al., in 2017 also reported the similar age group of couples had RSM,^14^,^21^ besides no statistical difference were calculated in the ages of the female and male carriers. However, frequency of miscarriages was found to be higher in male carriers as compare to female carriers (p-value<0.05). This highlighted the importance of genetic testing and genetic counseling even after single spontaneous miscarriage.

Moreover, no difference was found among SM and RSM when compared in different age groups. Though, highest number of miscarriages was found between the age of 26 and 30 years. This finding indicates the capability of women’s body is good enough at younger age, and struggle against the abnormal fetal development and results in miscarriage.^22^,^23^

In future pregnancies the probability of healthy child birth depends on the chromosome number and the type of rearrangement found among the couple. When one partner has the structural chromosomal abnormality it is highly recommended to perform embryonic karyotyping by amniocentesis, or chorionic villus sampling to avoid the possible chromosomal abnormality among their offspring

RSM persists as to be an exigent reproductive event for the family and physician, and cytogenetics can be helpful in screening heritable diseases. The results of this recent study re-emphasized the necessity of karyotyping as the first line of diagnostic test. This study would help physicians working in the region to realize the contribution of chromosomal abnormalities in repeated fetal loss. Probes should be designed to increase the efficacy of cytogenetic investigation.

### Author’s Contribution

**MIH:** Conception and design of study, acquisition of data, statistical analysis, manuscript writing.

**AK:** Conception and design, acquisition of data.

**AA:** Conception and design, manuscript editing, for important intellectual content.

**ES:** Conception and design, final approval of the manuscript version to be published.
